# Supplementary material for: Submarine landslide megablocks show half of Anak Krakatau island failed on December 22nd, 2018
Source: Nat Commun. 2021 May 14;12:2827. doi: 10.1038/s41467-021-22610-5 (PMC8121911; doi:10.1038/s41467-021-22610-5)
Supplement: Supplementary file 1 — Supplementary Information [file 41467_2021_22610_MOESM1_ESM.pdf]

Submarine landslide megablocks show half of Anak Krakatau island failed on December 22nd, 2018

Hunt, J.E.*[1], Tappin, D.R.[2,3], Watt, S.F.L.[4], Susilohadi, S. [5], Novellino, A.[2], Ebmeier, S.K.[6], Cassidy, M.[7], Engwell, S.L.[2], Grilli, S.T.[8], Hanif, M.[9], Priyanto, W.S.[9], Clare, M.A.[1], Abdurrachman, M.[10], Udrekh, U.[11]

[1] National Oceanography Centre, Southampton, UK
[2] British Geological Survey (BGS), Nottingham, UK
[3] University College London (UCL), London, UK.
[4] School of Geography, Earth and Environmental Sciences, University of Birmingham, Birmingham, UK
[5] Marine Geological Institute, Bandung, Indonesia
[6] School of Earth and Environment, University of Leeds, UK
[7] Department of Earth Sciences, University of Oxford, UK
[8] Department of Ocean Engineering, University of Rhode Island (URI), Narragansett, RI, USA.
[9] Research Center for Geotechnology, Indonesian Institute of Sciences (LIPI), Bandung, Indonesia
[10] Department of Geological Engineering, Institut Teknologi Bandung, Indonesia
[11] Badan Pengkajian dan Penerapan Teknologi, PTRRB-TPSA, DKI Jakarta, Java, Indonesia
* corresponding author: James.Hunt@noc.ac.uk

SUPPLEMENTARY INFORMATION

Supplementary Figure 1. Global distribution of collapse calderas above subduction zones. Points relate to mainland calderas less than 5 km from the coast and volcanic ocean island calderas (yellow), submarine calderas (blue), and post-collapse cones within caldera lakes (green).

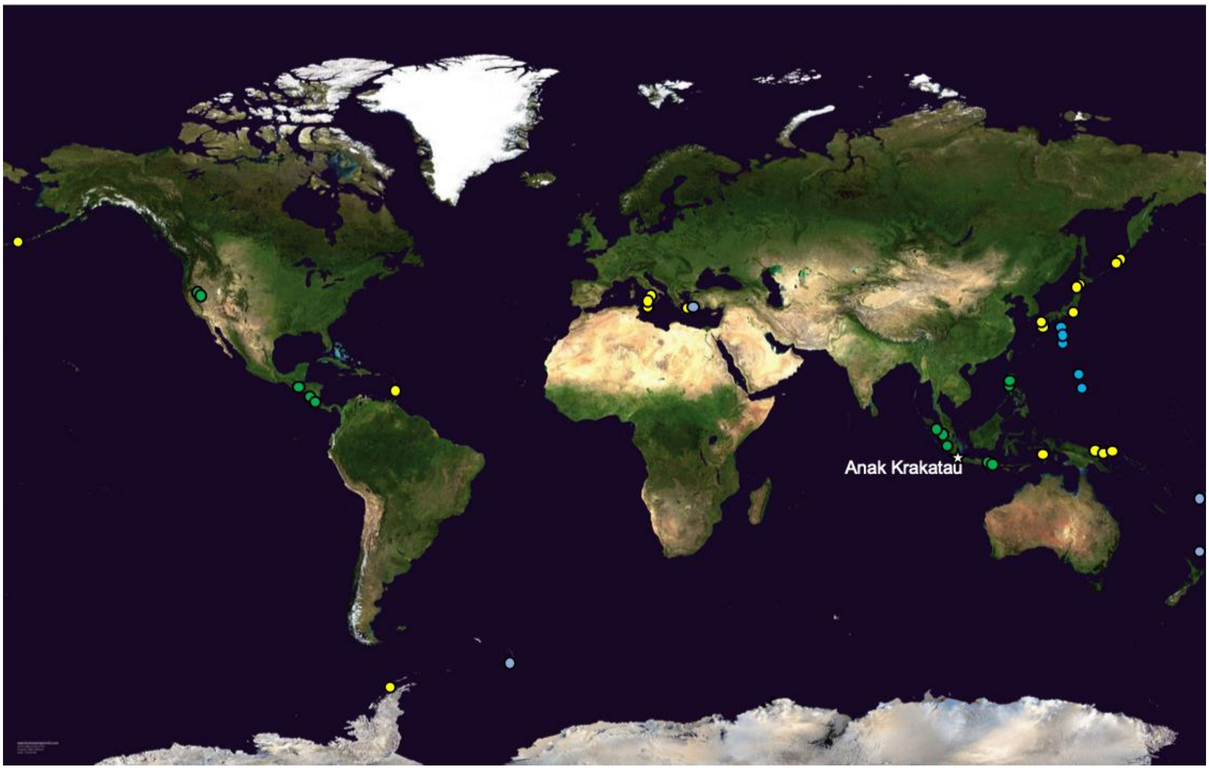

Supplementary Figure 2. Table showing the full available benchmarking data from the December 22nd, 2018 landslide-tsunami.

Type	Data	Description	References
Continuous direct monitoring	Seismicity and infrasound	Seismic and infrasound stations in Java and Sumatra record eruptions and landslide.	5, 22
	Satellite thermal data	Time-average discharge rates (TADR) derived from thermal data showing eruptive frequency and magnitude before and after the collapse.	5
	Atmospheric gas and ash emissions	Records of SO_2 mass fluxes and airborne ash mass concentrations from Himawari-8 satellite.	11
	Tide gauge data	Comprehensive tsunami observations including tide gauge records.	12
Discrete near-continuous monitoring	Flank Deformation	InSAR timeseries showing the deformation of SW flank.	5
	Island shape	Time series of island outline derived from visual images and SAR data from satellite constellation.	30
	Multispectral and synthetic aperture radar (SAR)	Pre- and post-collapse multispectral satellite and synthetic aperture radar (SAR) images from before (three days) and immediately after (eight hours) the event. We present the available satellite imagery including high-resolution SAR data from COSMO-SkyMed (CSK) on December 23rd, 2018, not included in earlier studies;	5, 22, 25, here
Pre- and post-event surveys	Bathymetry and topography	Pre-collapse (1990) basin bathymetry and island topography (2018) compared to new, recently acquired post-collapse high-resolution basin bathymetry (2019);	9,11, here
	Subseafloor seismic reflection	New pre- (2017) and post- (2019) collapse high-resolution seismic reflection data of the caldera basin.	Here
	Photographs	Photographs before during and after the collapse and eruptions.	Here
	Tsunami inundation survey	Comprehensive surveys of tsunami impact, and survivor accounts.	5, 14-16, 32-34

References in text.

Supplementary Figure 3. Sentinel-2 natural colour (bands 3, 2 and 1) images of the Krakatau island group from 09 Jun 2018 to 28 Jan 2019, showing volcanic activity leading up 22 Dec 2018 Anak Krakatau flank collapse and in early island recovery. Images downloaded from Sentinel Hub.

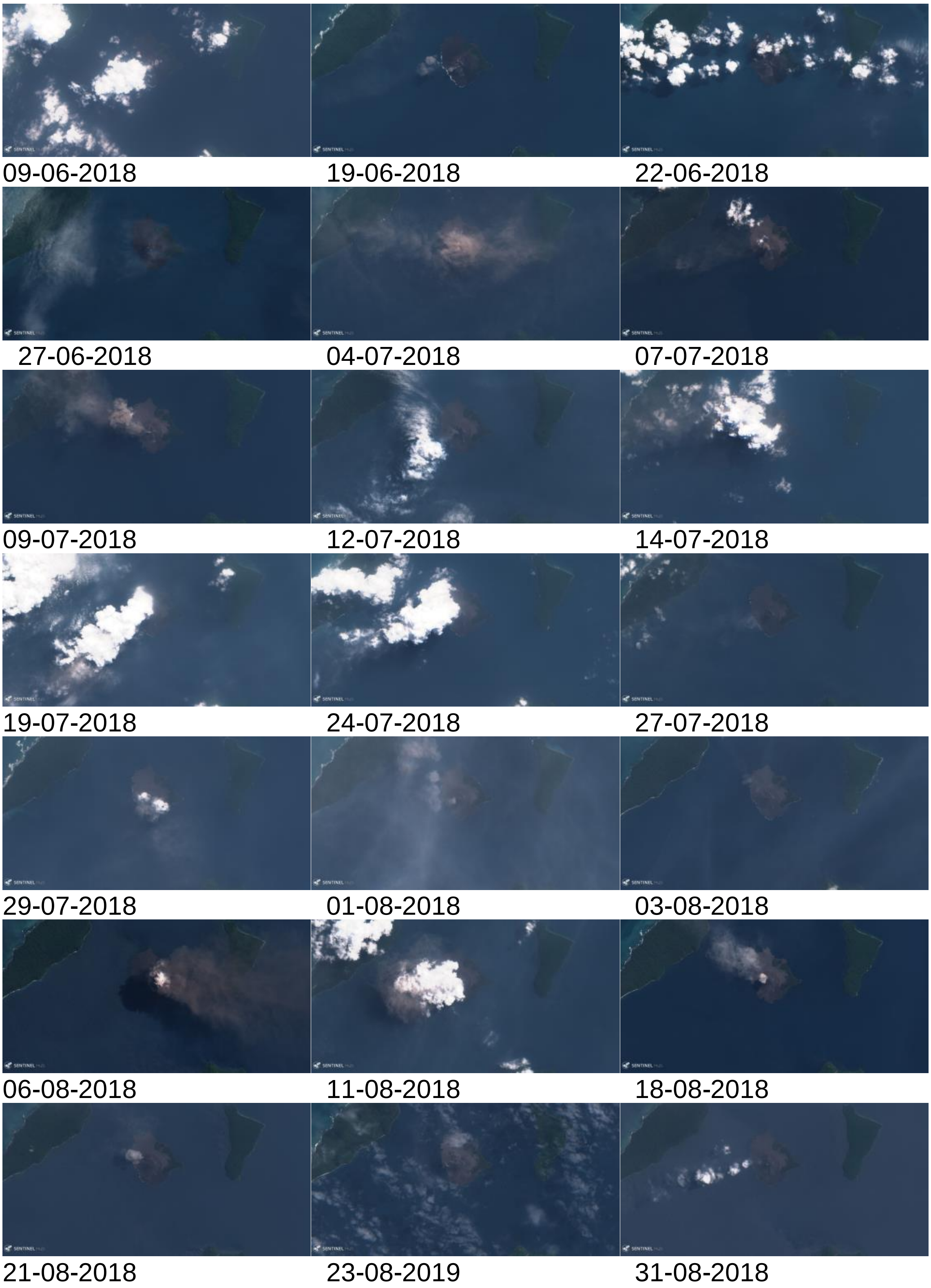

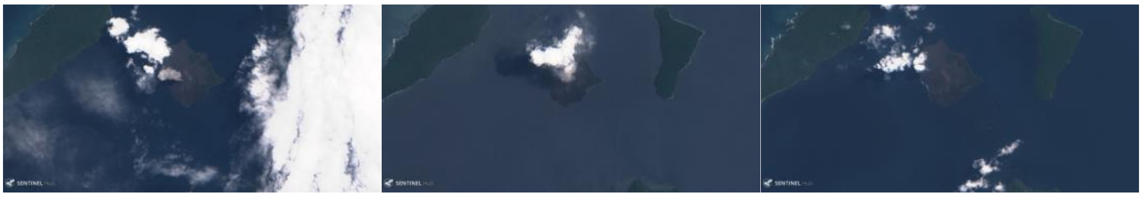

02-09-2018 05-09-2018 07-09-2018

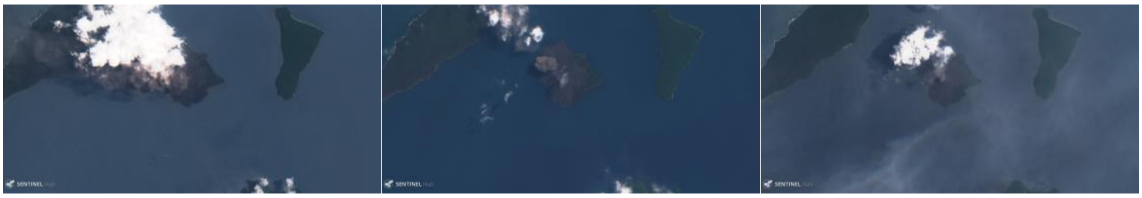

10-09-2018 12-09-2018 15-09-2018

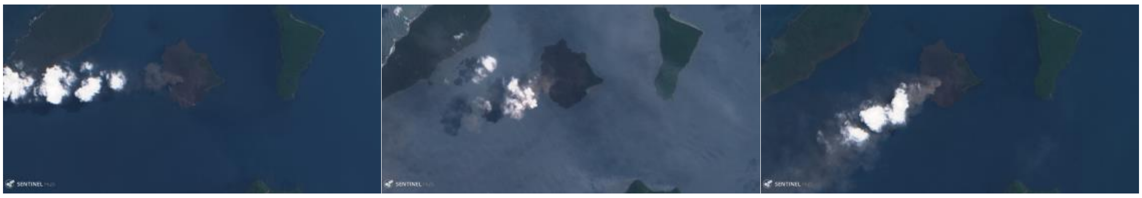

17-09-2018 20-09-2018 22-09-2018

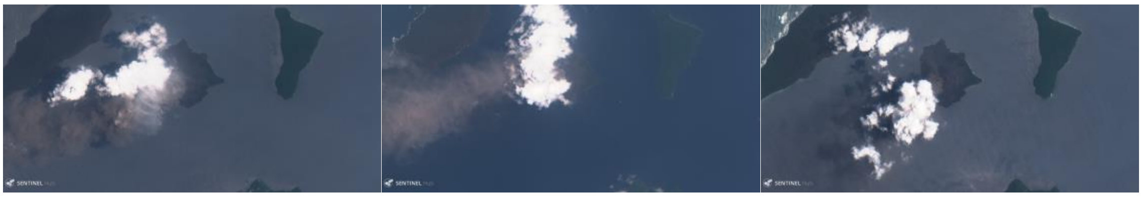

25-09-2018 27-09-2018 30-09-2018

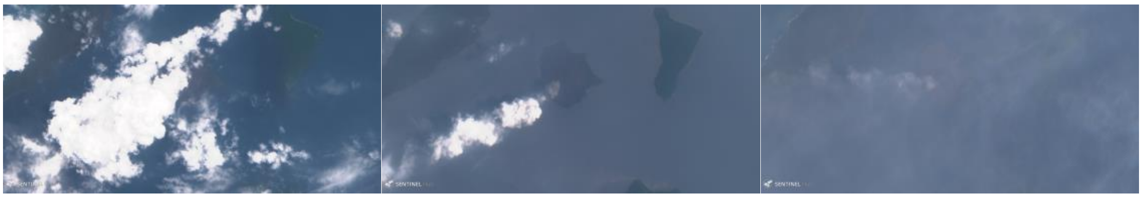

02-10-2018 05-10-2018 07-10-2018

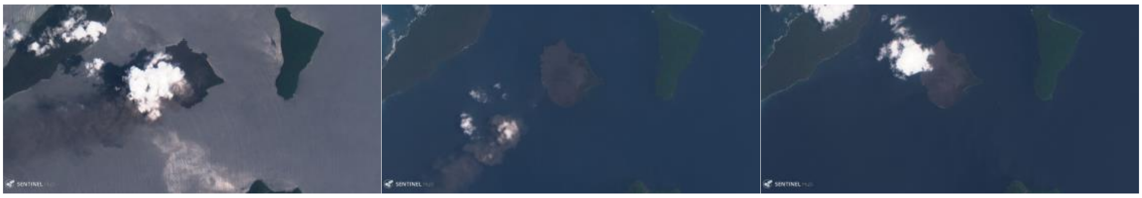

10-10-2018 12-10-2018 17-10-2018

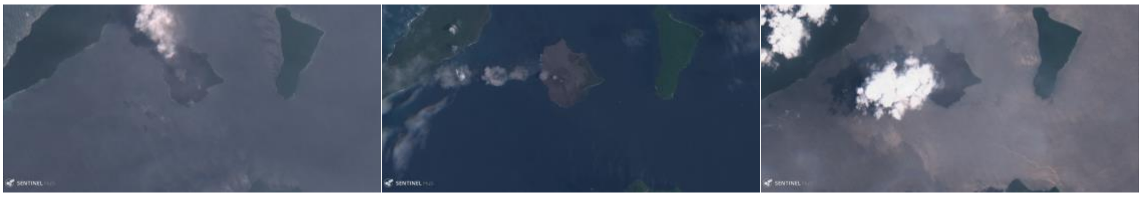

20-10-2018 22-10-2018 25-10-2018

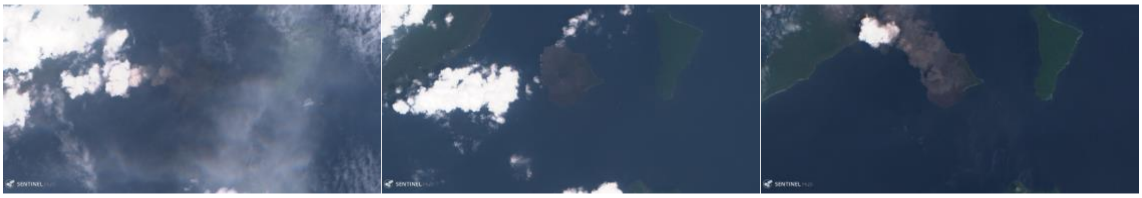

27-10-2018 01-11-2018 06-11-2018

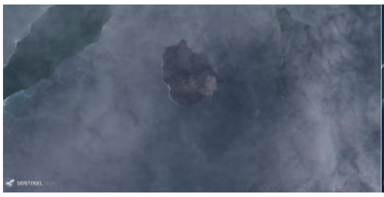
14-11-2018

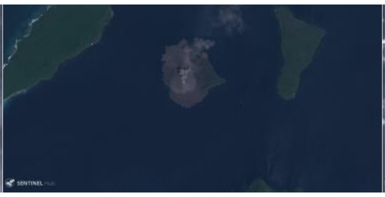
16-11-2018

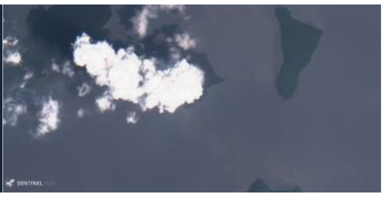
19-11-2018

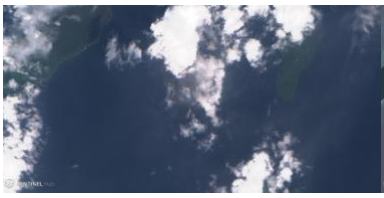
21-11-2018

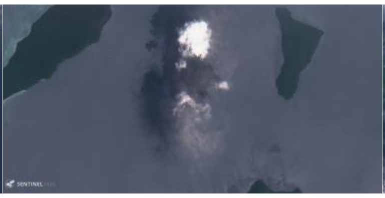
24-11-2018

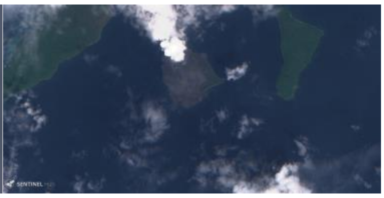
26-11-2018

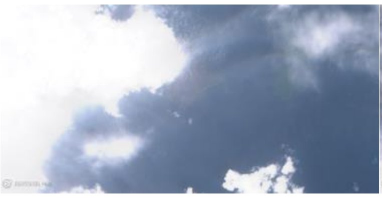
06-12-2018

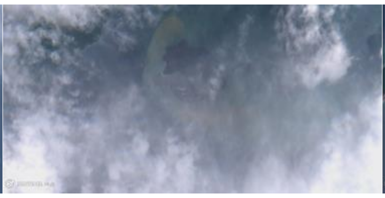
29-12-2018

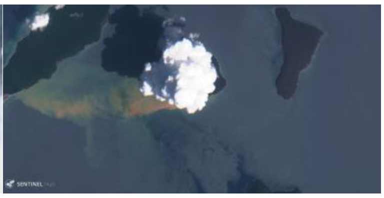
08-01-2019

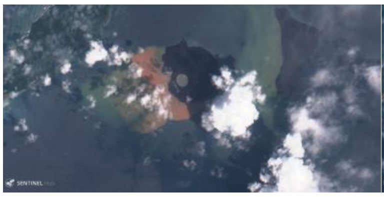
13-01-2019

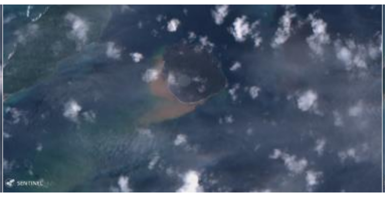
15-01-2019

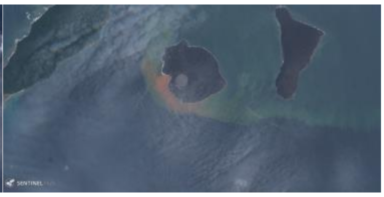
23-01-2019

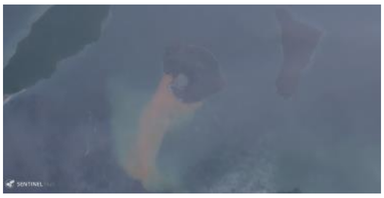
28-01-2019

Supplementary Figure 4. Sentinel-2 false colour (bands 4, 3 and 2) images of the Krakatau island group from 09 Jun 2018 to 29 Dec 2018, showing volcanic activity leading up 22 Dec 2018 Anak Krakatau flank collapse and in early island recovery. Images downloaded from Sentinel Hub.

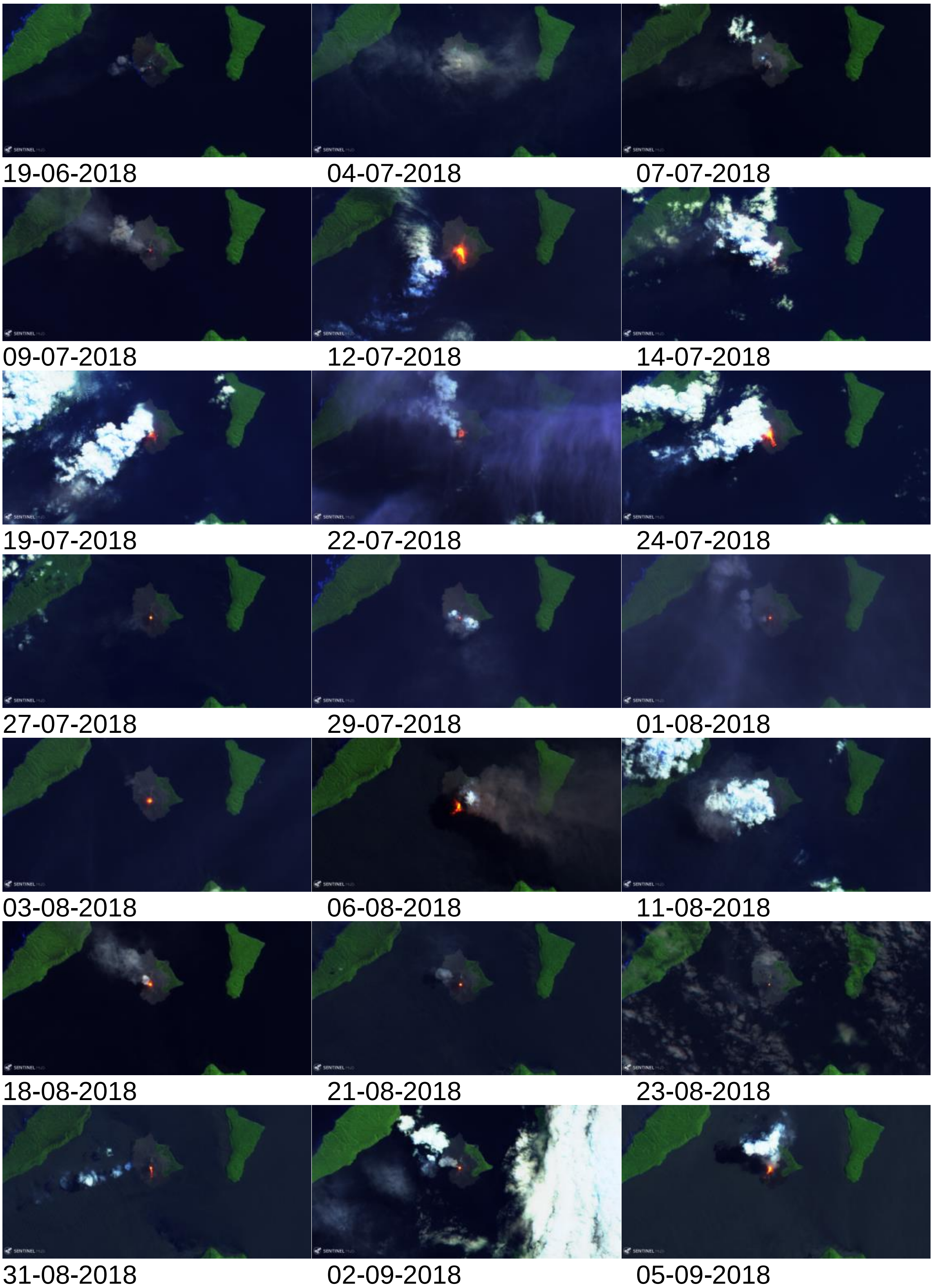

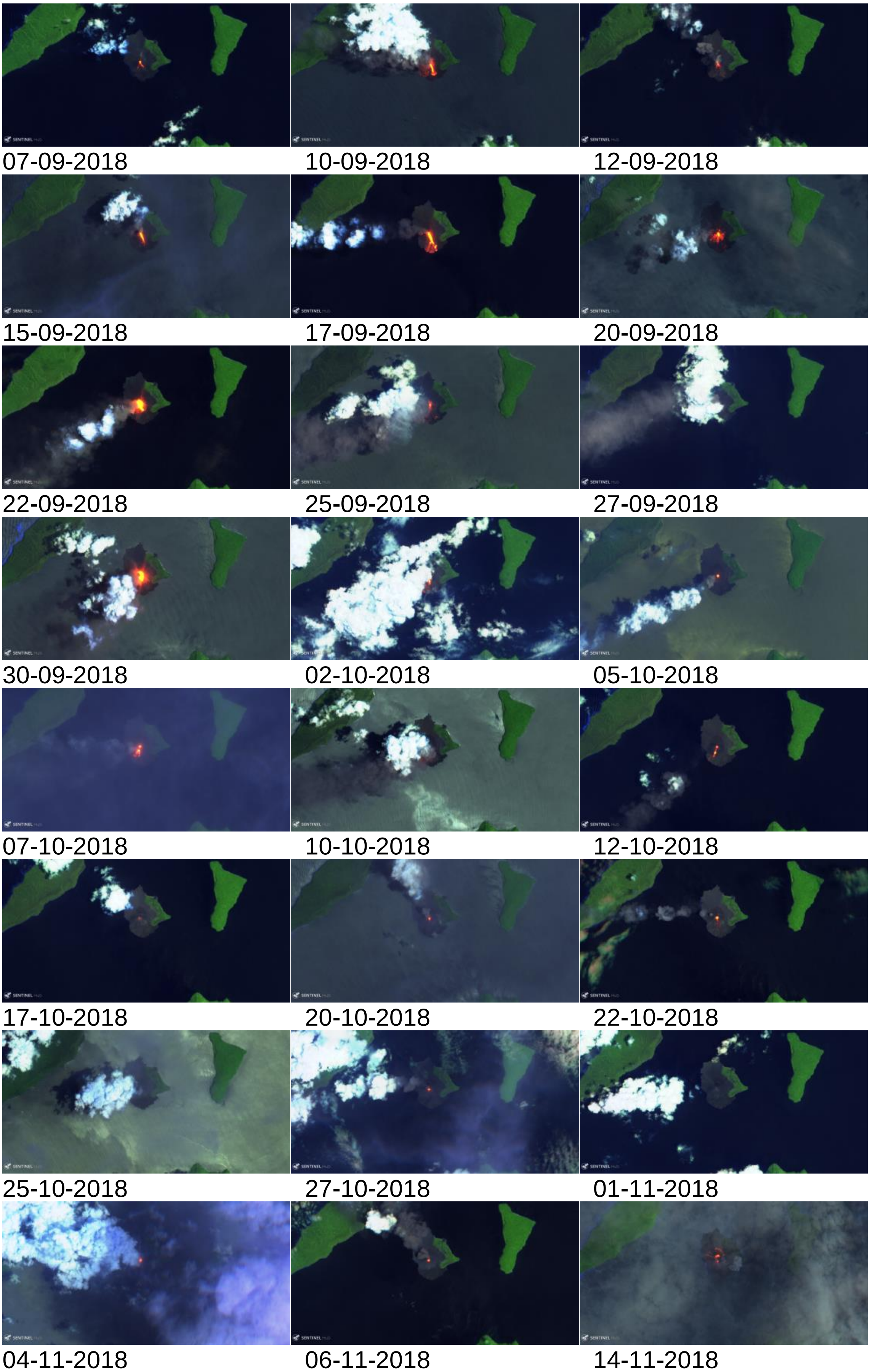
07-09-2018
10-09-2018
12-09-2018
15-09-2018
17-09-2018
20-09-2018
22-09-2018
25-09-2018
27-09-2018
30-09-2018
02-10-2018
05-10-2018
07-10-2018
10-10-2018
12-10-2018
17-10-2018
20-10-2018
22-10-2018
25-10-2018
27-10-2018
01-11-2018
04-11-2018
06-11-2018
14-11-2018

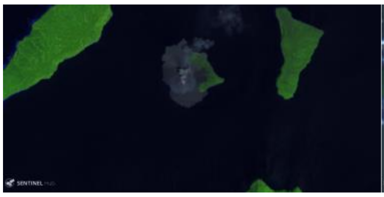
16-11-2018

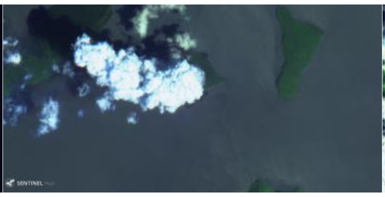
19-11-2018

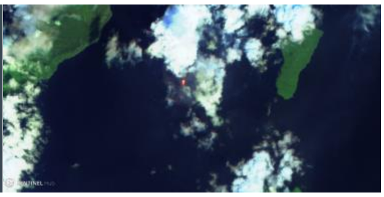
21-11-2018

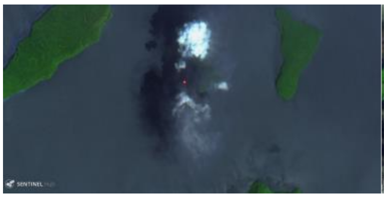
24-11-2018

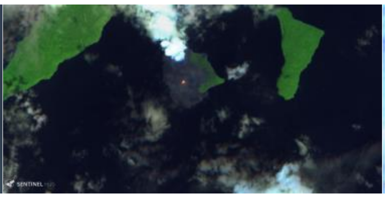
26-11-2018

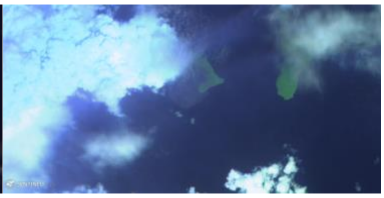
06-12-2018

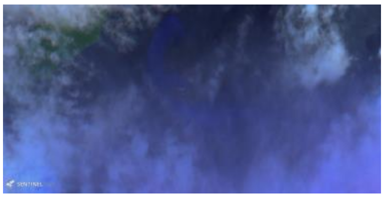
29-12-2018

Supplementary Figure 5. Photographs of Anak Krakatau before and after the December 22nd 2018 landslide showing the full extent of the subaerial failure. A) Image of Anak Krakatau on 05-08-18 showing the location of lavas build up on the SW flank and location of the future failure plain. B) Image of Anak Krakatau on 10-01-19 showing the subaerial results of the collapse of the SW flank of Anak Krakatau. We show the interpreted failure plain following the line of the ridhe representing a period collapsed crater. Images used with permission, with credits to Oystein Lund Andersen (S5A) and James Reynolds (S5B).

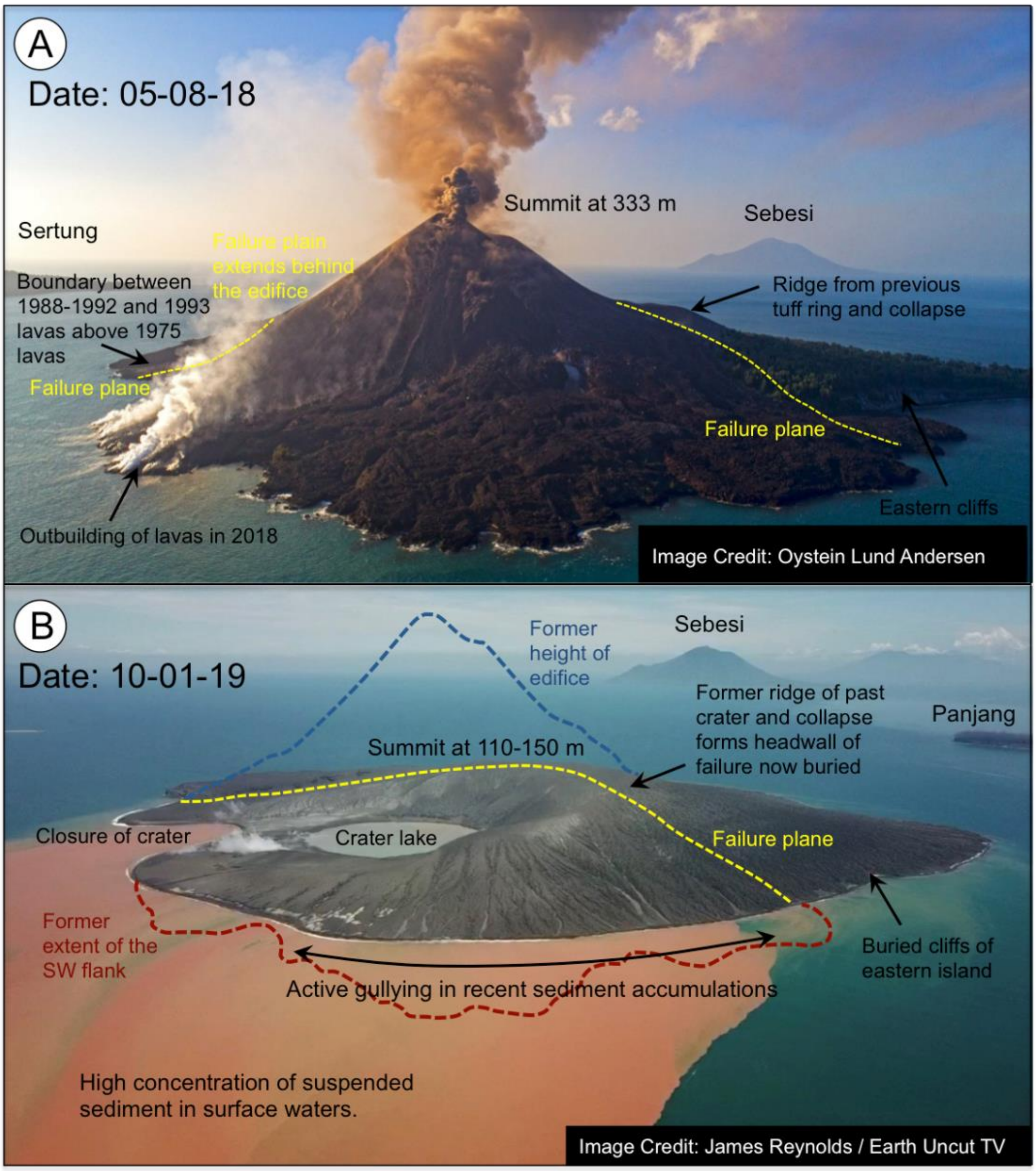

Supplementary Figure 6. Ninety years of volcanic activity of Anak Krakatau from 1927 to 2017.

Date*	Activity	Ref(s)
July 1927	Gas bubbles appeared within the caldera of Krakatau. Marks first activity since 1883 Krakatau eruption.	1, 2
29 Dec 1927	Submarine eruptions from within caldera sea. Eruption products build up. First phase of first period starts. Six ven develop and coalesce to form one.	1, 2
26 Jan 1928	Eruption products build up a cone that rises above sea lev as a long elongate island 175 m long and 3 m high (referre to as Anak Krakatau I).	1, 2
28 Jan 1928	Island has ‘eroded’ and disappeared. Eruptions form a second island 250 m long and 38 m high (referred to as Anak Krakatau II).	1, 2
25 Mar 1928	Explosions continue; second phase of first period.	1, 2
3 Jul 1928	Anak Krakatau II slides into the deep basin with complete disappearance of the island.	1, 2
6 Jul 1928	Explosions, marking third phase of first period.	1, 2
25 Aug 1928	Explosive activity, marking fourth phase of first period.	1, 2
19 Sep 1928	Explosive detonations.	1, 2
4 Nov 1928	Explosions, marking fifth phase of first period.	1, 2
11 Dec 1928	Explosive activity, marking sixth phase of first period.	1, 2
12 Jan 1929	Explosions, marking seventh phase of first period.	1, 2
6 Mar 1929	Explosions, marking eighth phase of first period.	1, 2
8 Jun 1929	Explosions, marking ninth phase of first period.	1, 2
25 Jul 1929	Explosions, marking tenth phase of first period.	1, 2
19 Sep 1929	Explosions, marking eleventh phase of first period.	1, 2
7 Dec 1929	Explosions move 600 m to SW within caldera to 250 m water depth, marking twelve phase of first period.	1, 2
4 Jan 1930	Explosions, marking thirteenth phase of first period.	1, 2
10 Mar 1930	Explosions, marking fourteenth phase of first period.	1, 2
30 Apr 1930	Explosions, marking fifteenth phase of first period.	1, 2
2 Jun 1930	Explosions, marking sixteenth phase of first period. On 8 Jun 1930 a new island emerged above sea level. On 14 Ju 1930 the island rose to 31 m with ash columns. On 25 Jun 1930 a crater rim emerged above sea level on all sides. Island reaches 50 m height and 375 m length.	1, 2
9 Aug 1930	A second part of the sixteenth phase is marked by destruction of the island by submarine eruptions.	1, 2
12 Aug 1930	New island emerges (referred to as Anak Krakatau IV).	1, 2
23 Sep 1931	Explosions and ash clouds rise 2.4 km high, marking the first phase of the second period with ash and lapilli fallout. The island is 47.2 m high and 1150 m long, with a crater 3 m wide.	1, 2
5 Nov 1931	Explosions, marking second phase of second period.	1, 2
5 Dec 1931	Explosions, marking third phase of second period.	1, 2
12 Feb 1932	Explosions, marking fourth phase of second period.	1, 2

Date*	Activity	Ref(s)
14 Nov 1932	Explosions, marking first phase of third period.	1, 2
16 Jan 1933	Explosions, marking second phase of third period. The island has grown to 96.9 m. On 1 May 1933 phreatomagmatic activity occurs with surges.	1, 2
10 Jun 1933	Explosions, marking third phase of third period.	1, 2
5 Jul 1933	Explosions, marking fourth phase of third period.	1, 2
5 Sep 1933	Explosions, marking fifth phase of third period.	1, 2
10 Nov 1933	Explosions, marking sixth phase of third period.	1, 2
6 Jan 1934	Explosions, marking seventh phase of third period.	1, 2
March 1934	Explosions, marking eighth phase of third period.	1, 2
5 May 1934	Explosions, marking ninth phase of third period.	1, 2
7 Jun 1934	Explosions, marking tenth phase of third period. The heigh of the island has reduced to 88.5 m.	1, 2
4 Jan 1935	Explosions, marking first phase of fourth period.	1, 2
6 Feb 1935	Explosions, marking second phase of fourth period.	1, 2
25 May 1935	Explosions, marking third phase of fourth period. Height of the island was 63.3 m.	1, 2
Aug 1935	Between Aug 1935 and Aug 1936 the SW coast of the island receded in the NE direction. In Aug 1936 the centre has shifted 400 m and the crater filled with sand.	1
13 Oct 1936	Fifth period demarked by medium-sized eruptions up to 30 m above the crater.	1, 2
6 Aug 1937	First phase of sixth period with 2.6 km ash columns.	1, 2
17 Nov 1937	Second phase of sixth period demarked with small eruptio columns.	1, 2
4 Jul 1938	Explosions, marking first phase of seventh period.	1, 2
12 Sep 1938	Explosions, marking second phase of seventh period.	1, 2
2 Oct 1938	Explosions, marking third phase of seventh period.	1, 2
7 Nov 1938	Explosions, marking fourth phase of seventh period.	1, 2
8 Dec 1938	Explosions, marking fifth phase of seventh period.	1, 2
15 Jan 1939	Explosions, marking sixth phase of seventh period.	1, 2
20 Mar 1939	Explosions, marking seventh phase of seventh period. Tw small craters are formed.	1, 2
1 Jun 1939	Explosions, marking eighth phase of seventh period. Stron eruptions with ash column 4 km high.	1, 2
23 Sep 1939	Explosions, marking ninth phase of seventh period.	1, 2
13 Dec 1939	Explosions, marking tenth phase of seventh period.	1, 2
3 Feb 1940	Explosions, marking eleventh phase of seventh period.	1, 2
1 Mar 1940	Explosions, marking twelfth phase of seventh period.	1, 2
10 Jun 1940	Explosions, marking thirteenth phase of seventh period.	1, 2
28 Jan 1941	Explosions, marking eighth period with eruptions in the crater lake.	1, 2
29 Jan 1942	Eruptions in the crater lake.	1, 2
1943	Probably active.	1, 2, 3
1944	Probably active.	1, 2
1945	Eruptions in the crater lake.	1, 2

Date*	Activity	Ref(s)
25 Jul 1946	Eruptions in crater lake.	1, 2
Jan 1947	Eruption clouds observed.	1, 2
Apr 1947	Eruptions in crater lake.	1, 2
12 May 1949	Eruptions in crater lake, with mud eruptions 120 m high. In Jun 1949, SW wall of the crater was ‘annihilated’ by ‘wave erosion’. The crater lake had a crescent shape.	1, 2
3 Jul 1950	Ash eruptions with explosions in the crater.	1, 4
10 Oct 1952	A 3 m-thick ash layer deposited on the island.	6
21 Sep 1953	Eruptions produce a column 3 km high.	4
25 Oct 1953	Explosive eruptions with ash clouds 4 km high.	4
2 Oct 1958	Detonations.	4
23 Oct 1958	Detonations.	4
5 Nov 1958	Three small ash eruptions.	4
4 Feb 1959	Ash eruption.	4
8 Jun 1959	Ash eruption with cloud 1.5 km high.	4
1950 to 1960	Topographical changes transform the island from a crescent, while crater lake has disappeared with lava flows extending across the crater floor.	1, 2, 5
2 Jul 1965	Eruptions, details uncertain.	7
26 Jun 1972	Large eruption plume on 26 June 1972.	7
16 Oct 1972	Underwater eruption seen along south coast.	7
27 Mar 1975		7
10 Jul 1978	Volcano ejected a column of incandescent material, visible from the W Java coast.	8
15 Jul 1979	Bombs, lapilli, and ash were ejected, rising 200 m and covering the area within about 700 m of the crater. Lava flowed 450 m W, reaching the coast.	9
15 Mar 1980	Incandescent material rose 200 m above the vent.	10
20 Oct 1981	Eruption column typically to 400-600 m, but occasionally to 2 km in height.	11
14 Feb 1988	Ash emission with crater detonations.	12
16 Mar 1988	Two lava flows from new crater.	12
7 Nov 1992	Lava flows and incandescent tephra.	13
11 Nov 1992	Incandescent tephra ejection; lava reaches sea. Strombolian explosions with ash columns up to 400 m by Jan-Feb 1993.	13, 14
19 Mar 1994	Ash clouds and tephra ejection.	15
Jan-Mar 1995	Ash plumes daily up to 500 m above the summit.	16
29 Sep 1996	A plume up to 3.7 km high. Lavas flows and ash explosions.	17
16 Mar 1997	Ash eruptions, with column up to 7 km.	18
5 Feb 1999	Ash eruptions, with column up to 1 km. Strombolian eruptions continue through Aug 1999.	19
29 May 2000	Small ash plumes.	20
21 Jul 2001	Ash eruptions with column reported to 6.1 km. Activity continues into Sep 2001 and through to Mar 2003.	21, 22

Date*	Activity	Ref(s)
23 Oct 2007	Minor eruptions.	23
25 Mar 2009	Variable minor eruptions through to May 2009.	24
25 Oct 2010	Significant eruption column and explosive activity.	25
31 Jul 2011	Magma ascent recorded followed by pyroclasic acitivt Fire fountaining recorded to 11 m heights. Activity continues through Sep 2011.	25
12 Nov 2011	Mild Strombolian activity.	25
Sep 2012	Continued acitivty with ash fall reaching Sumatra. Lav flows reach the sea.	26
Aug 2015	Diffuse white plumes. Continued low-level activity through 2015 and early 2016.	27
17 Feb 2017	Large lava flow that reaches the sea on the SE flank.	28
19 Jun 2018	Renewed explosive eruptions, ejecta and lavas. Activity through Jun to Oct, Nov and then finally in De leading to failure of SW flank of Anak Krakatau.	29

* Date at the start of activity

References out of text:

[1] van Padang, M.N., 1951. Catalogue of the active volcanoes of Indonesia. *International Volcanological Association*.

[2] Hedervari, P., 1986. Catalog of submarine volcanoes and hydrological phenomena associated with volcanic events, January 1, 1900 to December 31, 1959. Report SE-42, World Data Centre, A for Solid Earth Geophysics, pp.1-35.

[3] Kusumadinata, K., 1979. Catalogue of references on Indonesian volcanoes with eruiptions in historical time. the *Directorate of Volcanology Indonesia*.

[4] Suryo, I., 1978. Volcanic phenomena during the year 1960. *Bulletin of the Volcanological Survey of Indonesia*, **1-3**.

[5] Zen, M.T. and Hadikusumo, D., 1964. Preliminary report on the 1963 eruption of Mt. Agung in Bali (Indonesia). *Bulletin Volcanologique*, **27**(1), 269-299.

[6] Decker, R.W. and Hadikusumo, D., 1961. Results of the 1960 expedition to Krakatau. *Journal of Geophysical Research*, **66**(10), 3497-3511.

[7] Global Volcanism Program, Smithsonian Institute.

[8] Global Volcanism Program, 1978. Report on Krakatau (Indonesia). In: Squires, D. (ed.), Scientific Event Alert Network Bulletin, 3:7. Smithsonian Institution. https://doi.org/10.5479/si.GVP.SEAN197807-262000.

[9] Global Volcanism Program, 1979. Report on Krakatau (Indonesia). In: Squires, D. (ed.), Scientific Event Alert Network Bulletin, 4:7. Smithsonian Institution. https://doi.org/10.5479/si.GVP.SEAN197907-262000.

[10] Global Volcanism Program, 1980. Report on Krakatau (Indonesia). In: Squires, D. (ed.), Scientific Event Alert Network Bulletin, 5:4. Smithsonian Institution. https://doi.org/10.5479/si.GVP.SEAN198004-262000.

[11] Global Volcanism Program, 1981. Report on Krakatau (Indonesia). In: McClelland, L. (ed.), Scientific Event Alert Network Bulletin, 6:10. Smithsonian Institution. https://doi.org/10.5479/si.GVP.SEAN198110-262000.

[12] Global Volcanism Program, 1988. Report on Krakatau (Indonesia). In: McClelland, L. (ed.), Scientific Event Alert Network Bulletin, 13:2. Smithsonian Institution. https://doi.org/10.5479/si.GVP.SEAN198802-262000.

[13] Global Volcanism Program, 1992. Report on Krakatau (Indonesia). In: McClelland, L. (ed.), Bulletin of the Global Volcanism Network, 17:10. Smithsonian Institution. https://doi.org/10.5479/si.GVP.BGVN199210-262000.

[14] Global Volcanism Program, 1993. Report on Krakatau (Indonesia). In: McClelland, L. (ed.), Bulletin of the Global Volcanism Network, 18:1. Smithsonian Institution. https://doi.org/10.5479/si.GVP.BGVN199301-262000.
[15] Global Volcanism Program, 1994. Report on Krakatau (Indonesia). In: Wunderman, R. (ed.), Bulletin of the Global Volcanism Network, 19:4. Smithsonian Institution. https://doi.org/10.5479/si.GVP.BGVN199404-262000.
[16] Global Volcanism Program, 1995. Report on Krakatau (Indonesia). In: Wunderman, R. (ed.), Bulletin of the Global Volcanism Network, 20:3. Smithsonian Institution. https://doi.org/10.5479/si.GVP.BGVN199503-262000.
[17] Global Volcanism Program, 1996. Report on Krakatau (Indonesia). In: Wunderman, R. (ed.), Bulletin of the Global Volcanism Network, 21:9. Smithsonian Institution. https://doi.org/10.5479/si.GVP.BGVN199609-262000.
[18] Global Volcanism Program, 1997. Report on Krakatau (Indonesia). In: Wunderman, R. (ed.), Bulletin of the Global Volcanism Network, 22:7. Smithsonian Institution. https://doi.org/10.5479/si.GVP.BGVN199707-262000.
[19] Global Volcanism Program, 1999. Report on Krakatau (Indonesia). In: Wunderman, R. (ed.), Bulletin of the Global Volcanism Network, 24:4. Smithsonian Institution. https://doi.org/10.5479/si.GVP.BGVN199904-262000.
[20] Global Volcanism Program, 2000. Report on Krakatau (Indonesia). In: Wunderman, R. (ed.), Bulletin of the Global Volcanism Network, 25:5. Smithsonian Institution. https://doi.org/10.5479/si.GVP.BGVN200005-262000.
[21] Global Volcanism Program, 2001. Report on Krakatau (Indonesia). In: Wunderman, R. (ed.), Bulletin of the Global Volcanism Network, 26:1. Smithsonian Institution. https://doi.org/10.5479/si.GVP.BGVN200101-262000.
[22] Global Volcanism Program, 2003. Report on Krakatau (Indonesia). In: Venzke, E. (ed.), Bulletin of the Global Volcanism Network, 28:3. Smithsonian Institution. https://doi.org/10.5479/si.GVP.BGVN200303-262000.
[23] Global Volcanism Program, 2007. Report on Krakatau (Indonesia). In: Wunderman, R. (ed.), Bulletin of the Global Volcanism Network, 32:9. Smithsonian Institution. https://doi.org/10.5479/si.GVP.BGVN200709-262000.
[24] Global Volcanism Program, 2009. Report on Krakatau (Indonesia). In: Wunderman, R. (ed.), Bulletin of the Global Volcanism Network, 34:5. Smithsonian Institution. https://doi.org/10.5479/si.GVP.BGVN200905-262000.
[25] Global Volcanism Program, 2011. Report on Krakatau (Indonesia). In: Wunderman, R. (ed.), Bulletin of the Global Volcanism Network, 36:8. Smithsonian Institution. https://doi.org/10.5479/si.GVP.BGVN201108-262000.
[26] Global Volcanism Program, 2012. Report on Krakatau (Indonesia). In: Wunderman, R. (ed.), Bulletin of the Global Volcanism Network, 37:12. Smithsonian Institution. https://doi.org/10.5479/si.GVP.BGVN201212-262000.
[27] Global Volcanism Program, 2015. Report on Krakatau (Indonesia). In: Venzke, E. (ed.), Bulletin of the Global Volcanism Network, 40:8. Smithsonian Institution. https://doi.org/10.5479/si.GVP.BGVN201508-262000.
[28] Global Volcanism Program, 2017. Report on Krakatau (Indonesia). In: Venzke, E. (ed.), Bulletin of the Global Volcanism Network, 42:9. Smithsonian Institution. https://doi.org/10.5479/si.GVP.BGVN201709-262000.
[29] Walter, T.R., Haghighi, M.H., Schneider, F.M., Coppola, D., Motagh, M., Saul, J., Babeyko, A., Dahm, T., Troll, V.R., Tilmann, F. and Heimann, S., 2019. Complex hazard cascade culminating in the Anak Krakatau sector collapse. *Nature Communications*, **10**(1), 1-11.

Supplementary Figure 7. Sentinel-2 images showing preconditioning events. False-colour images showing occurrence of lavas and Strombolian activity. True-colour images showing occurrence of fissures north of the crater highlighted by arrows. True-colour images showing occurrence of fumarole activity from fumarole deposits highlighted by arrows.

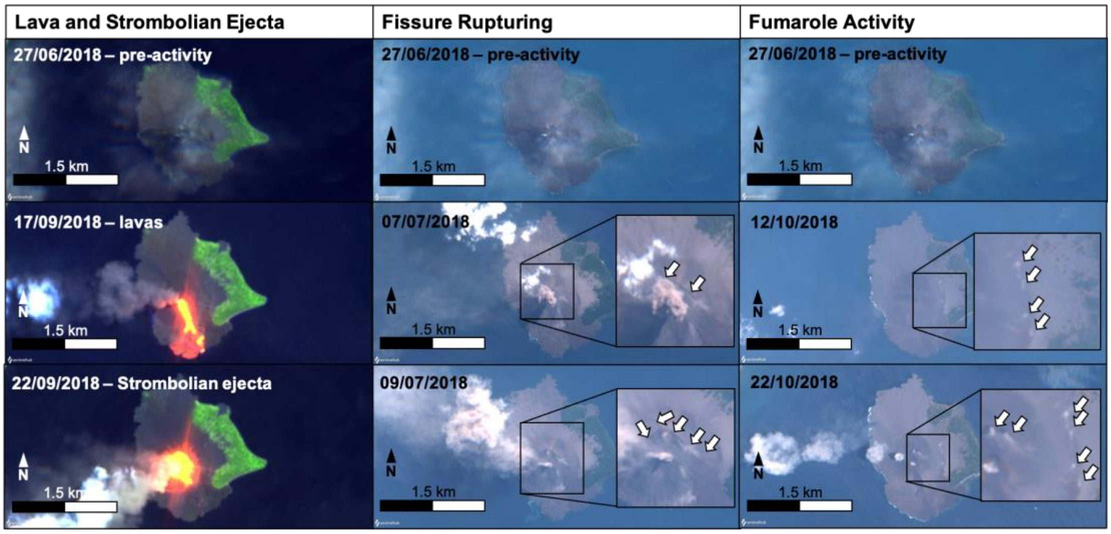

Supplementary Figure 8. Maps of pre- and post-event data collection cruise track-lines. A) Pre-event bathymetry (1990, white lines) and seismic reflection profiles (2017, yellow lines); B Post-event bathymetry (2019, closely-spaced pale red lines) and seismic reflection profiles (2019, bold red lines).

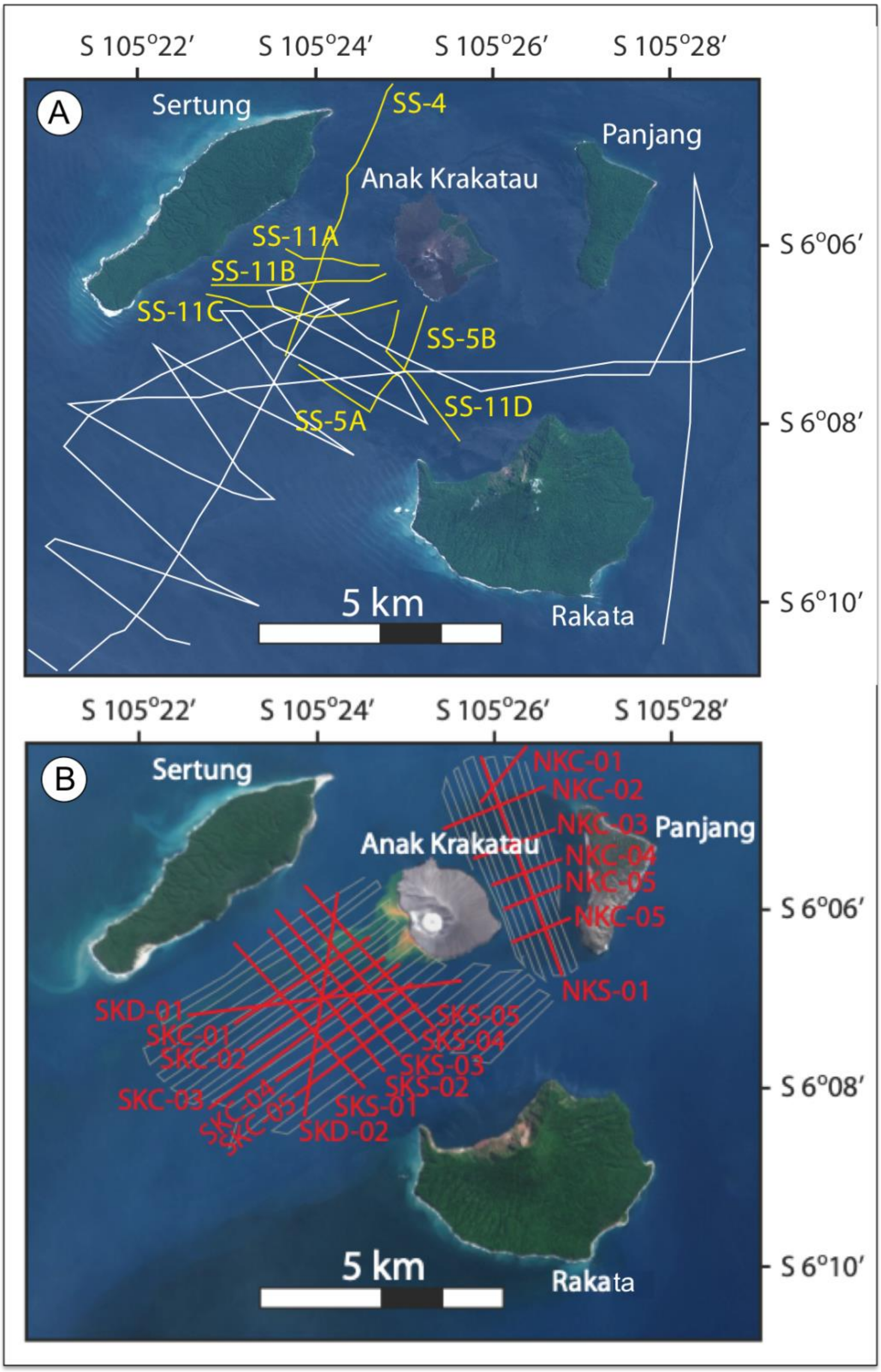

Supplementary Figure 9. Time series of satellite synthetic aperture radar images of Anak Krakatau before, during and after the December 22nd, 2018 flank collapse. A) Sentinel-1 SAR December 19th, 2018; B) Sentinel-1 SAR December 22nd, 2018 ; C) COSMO SkyMED SAR December 24th, 2018; D) ALOS-2 SAR December 24th, 2018; E) Sentinel-1 SAR December 25th, 2018; F) RADARSAT-2 SAR December 26th, 2018; G) Sentinel-1 SAR December 27th, 2018; H) Sentinel-1 SAR December 28th, 2018; I) TerraSAR-X December 28th, 2018; J) Sentinel-1 radar December 31st, 2018; K) Sentinel-1 radar January 2nd, 2019; and L) TerraSAR-X January 8th, 2019. Yellow dotted lines represent the interpretation of the failure plane. Red arrows represent the viewing angle of the satellite.

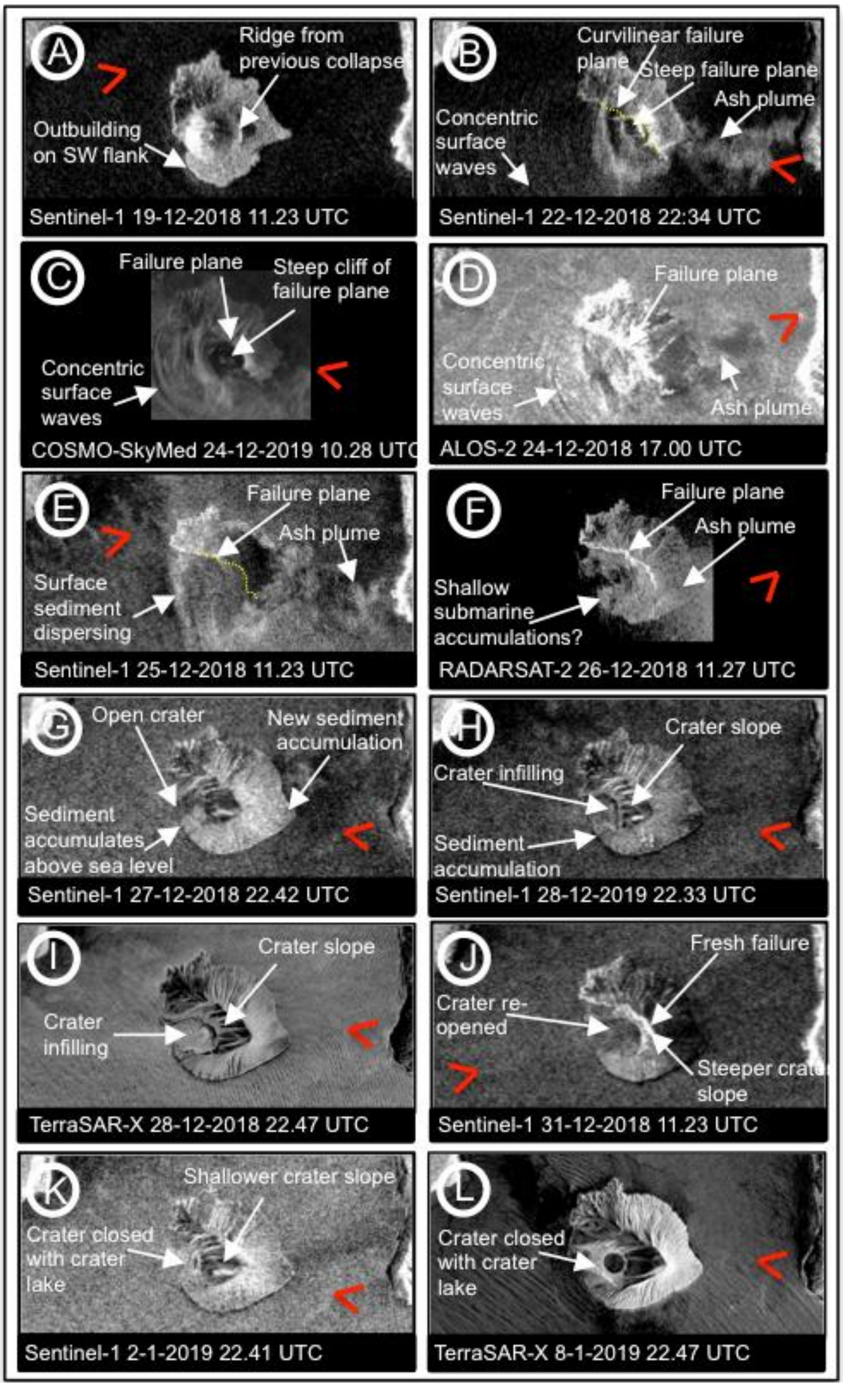

Supplementary Figure 10. Uninterpreted seismic reflection profiles used in figure 4: SS11B, SS11D, SS5B and SS 11C; those used in figure 6: SKC-03, SKC-02 and SKC-01; and those used in figure 7: SKS-01, SKS-02 and SKS-04.

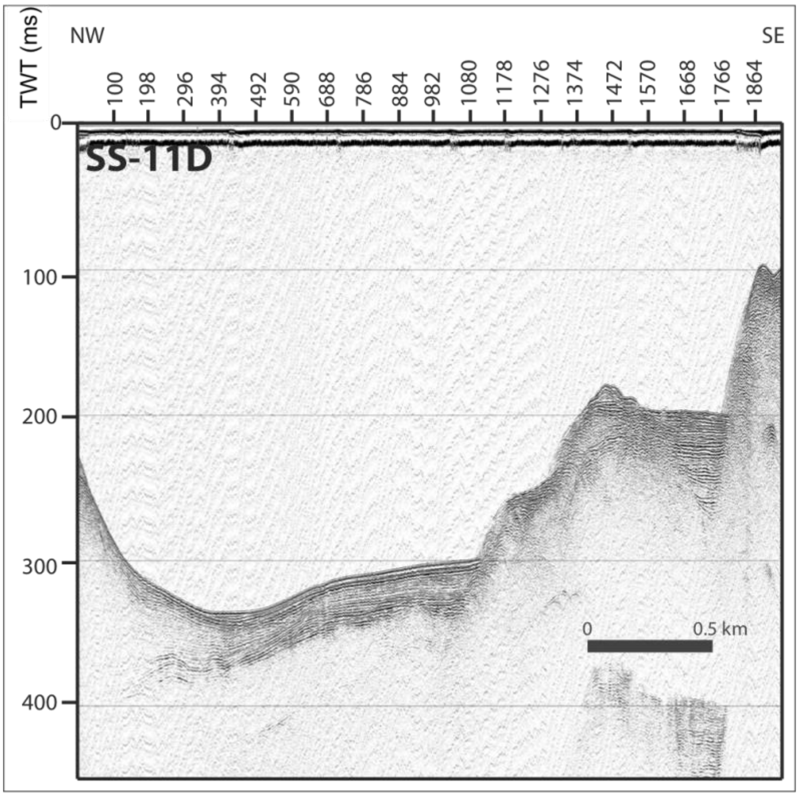

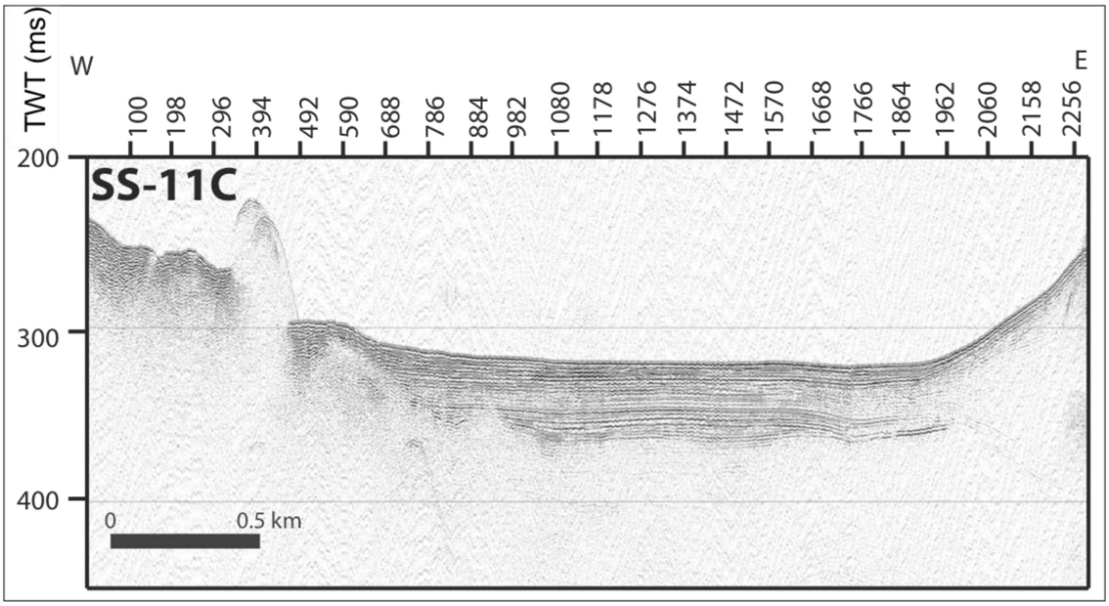

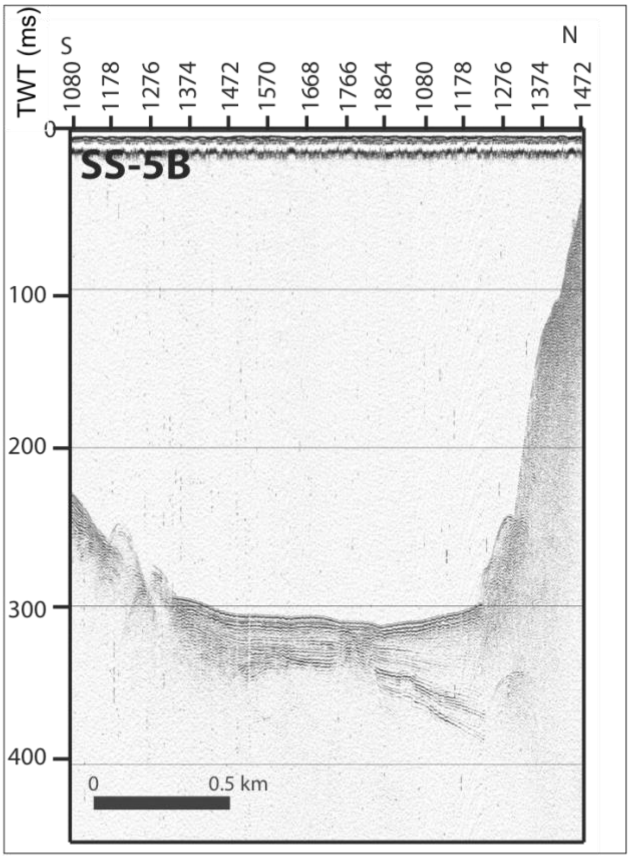
TWT (ms)
S
N
1080
1178
1276
1374
1472
1570
1668
1766
1864
1080
1178
1276
1374
1472
SS-5B
0
100
200
300
400
0
0.5 km

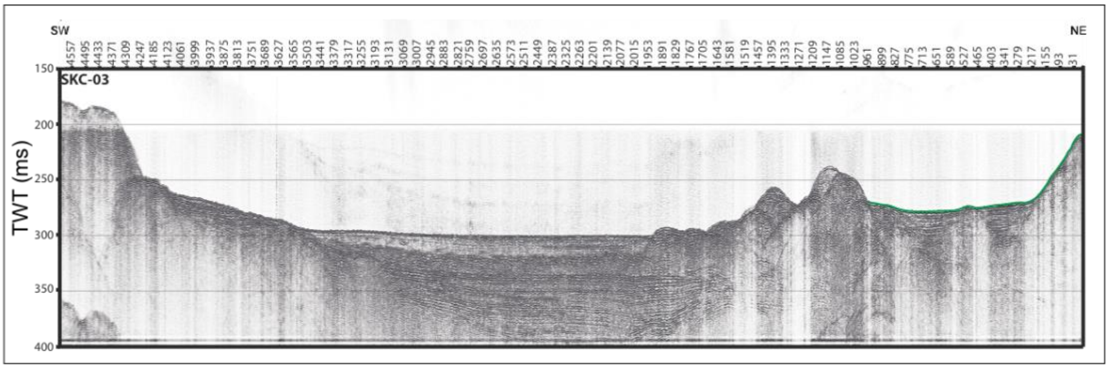
SW
NE
SKC-03
TWT (ms)
150
200
250
300
350
400

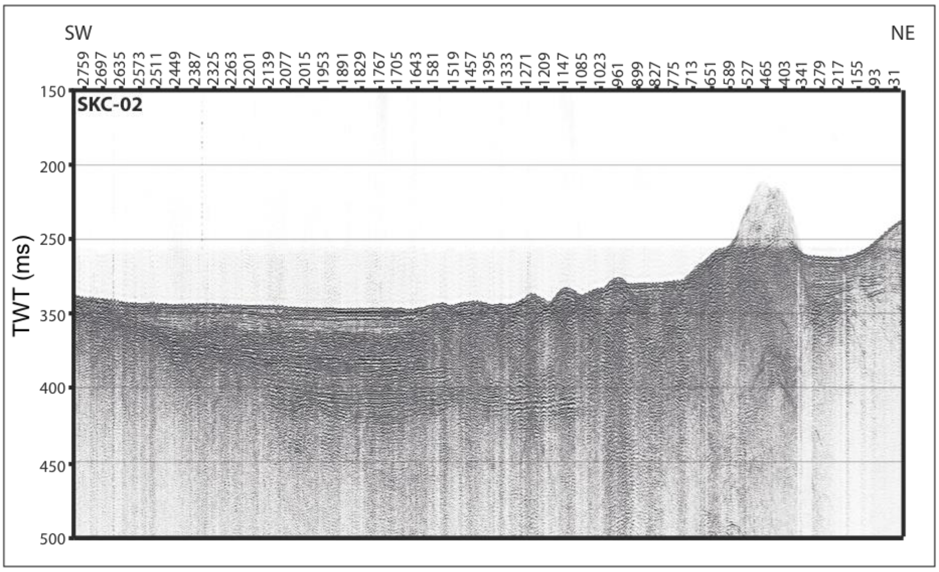
SW
NE
2759
2697
2635
2573
2511
2449
2387
2325
2263
2201
2139
2077
2015
1953
1891
1829
1767
1705
1643
1581
1519
1457
1395
1333
1271
1209
1147
1085
1023
961
899
827
775
713
651
589
527
465
403
341
279
217
155
93
31
SKC-02
TWT (ms)
150
200
250
350
400
450
500

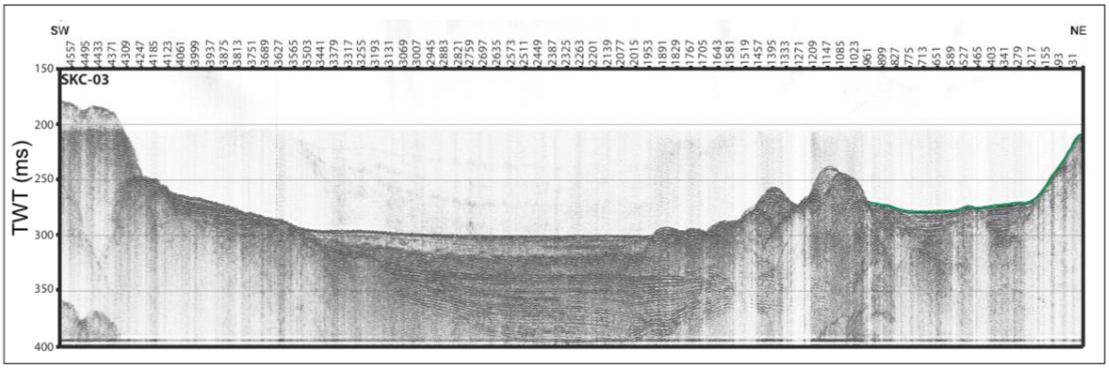
SW
NE
SKC-03
TWT (ms)
150
200
250
300
350
400

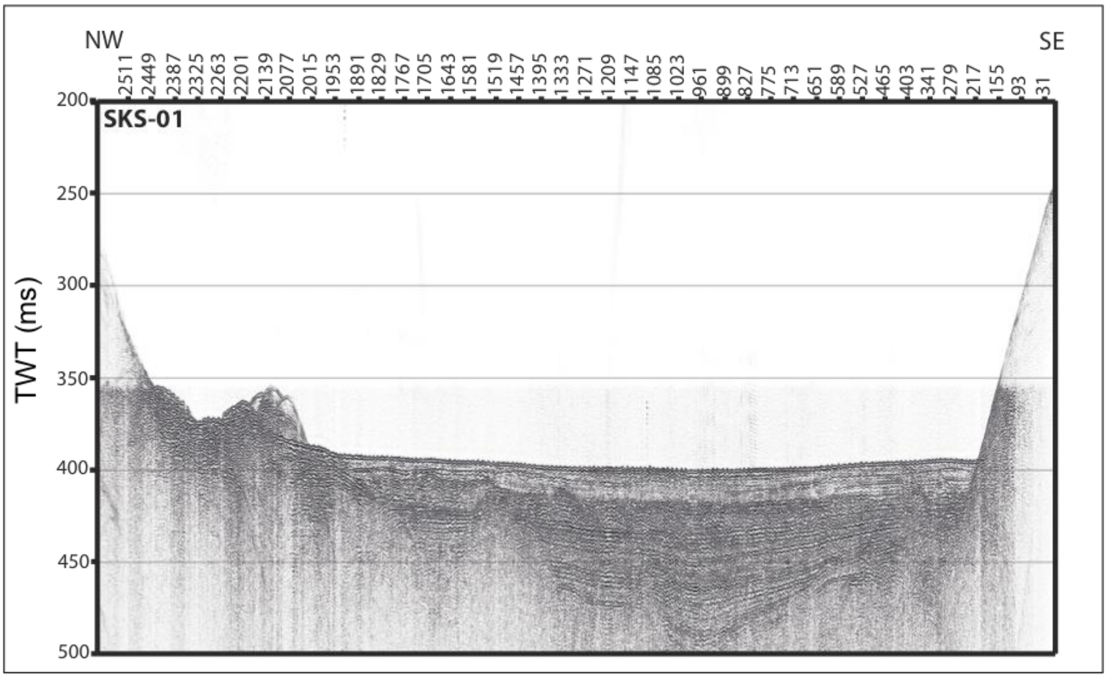
NW
SE
2511 2449 2387 2325 2263 2201 2139 2077 2015 1953 1891 1829 1767 1705 1643 1581 1519 1457 1395 1333 1271 1209 1147 1085 1023 961 899 827 775 713 651 589 527 465 403 341 279 217 155 93 31
SKS-01
TWT (ms)
200
250
300
350
400
450
500

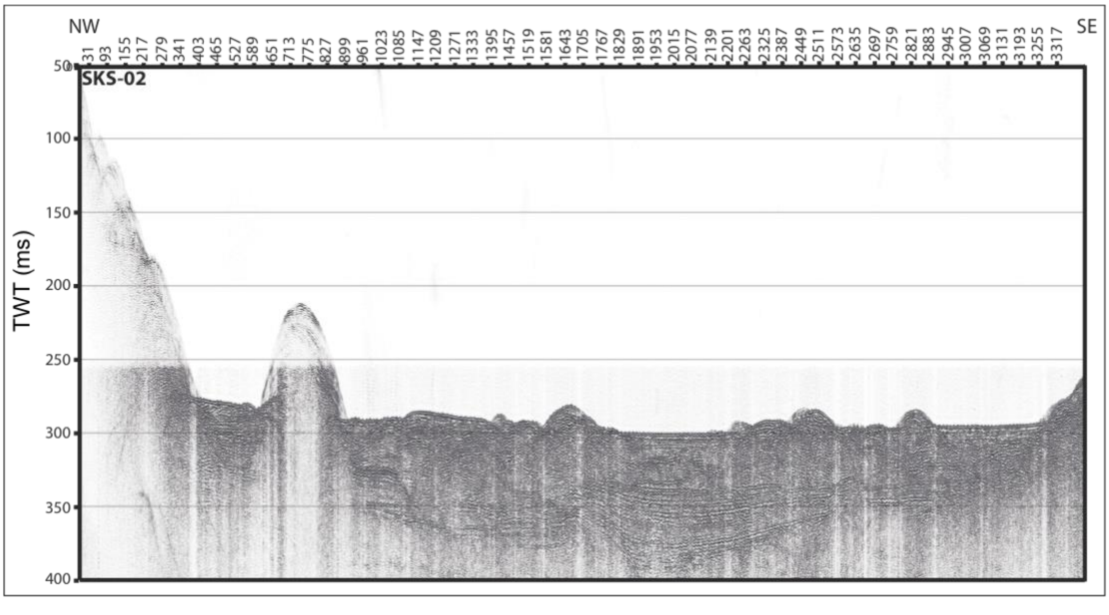
NW
SE
31 93 155 217 279 341 403 465 527 589 651 713 775 827 899 961 1023 1085 1147 1209 1271 1333 1395 1457 1519 1581 1643 1705 1767 1829 1891 1953 2015 2077 2139 2201 2263 2325 2387 2449 2511 2573 2635 2697 2759 2821 2883 2945 3007 3069 3131 3193 3255 3317
SKS-02
TWT (ms)
50
100
150
200
250
300
350
400

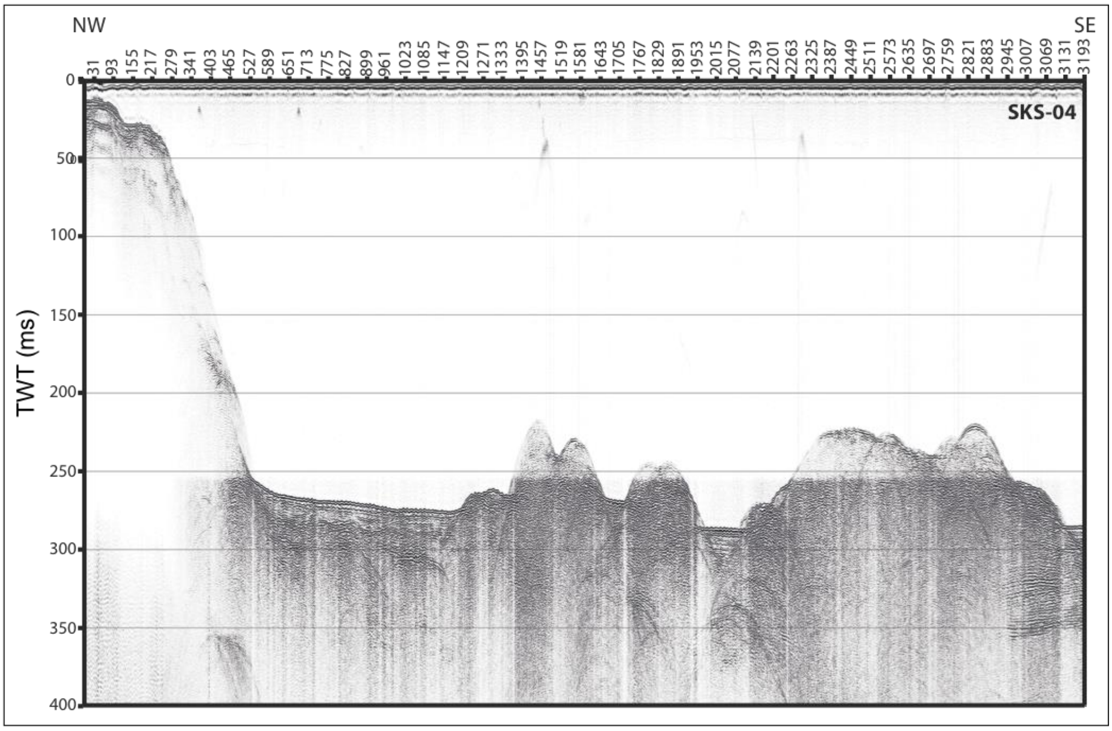
NW
SE
SKS-04
TWT (ms)
0
50
100
150
200
250
300
350
400
31
93
155
217
279
341
403
465
527
589
651
713
775
827
899
961
1023
1085
1147
1209
1271
1333
1395
1457
1519
1581
1643
1705
1767
1829
1891
1953
2015
2077
2139
2201
2263
2325
2387
2449
2511
2573
2635
2697
2759
2821
2883
2945
3007
3069
3131
3193

Supplementary Figure 11. Magnified regions of 2019 seismic reflection profiles showing the boundary between the landslide and secondary debris flow (now buried) from A) SKC-03; B) SKC-02; C) SKS-01; and D) SKS-02.

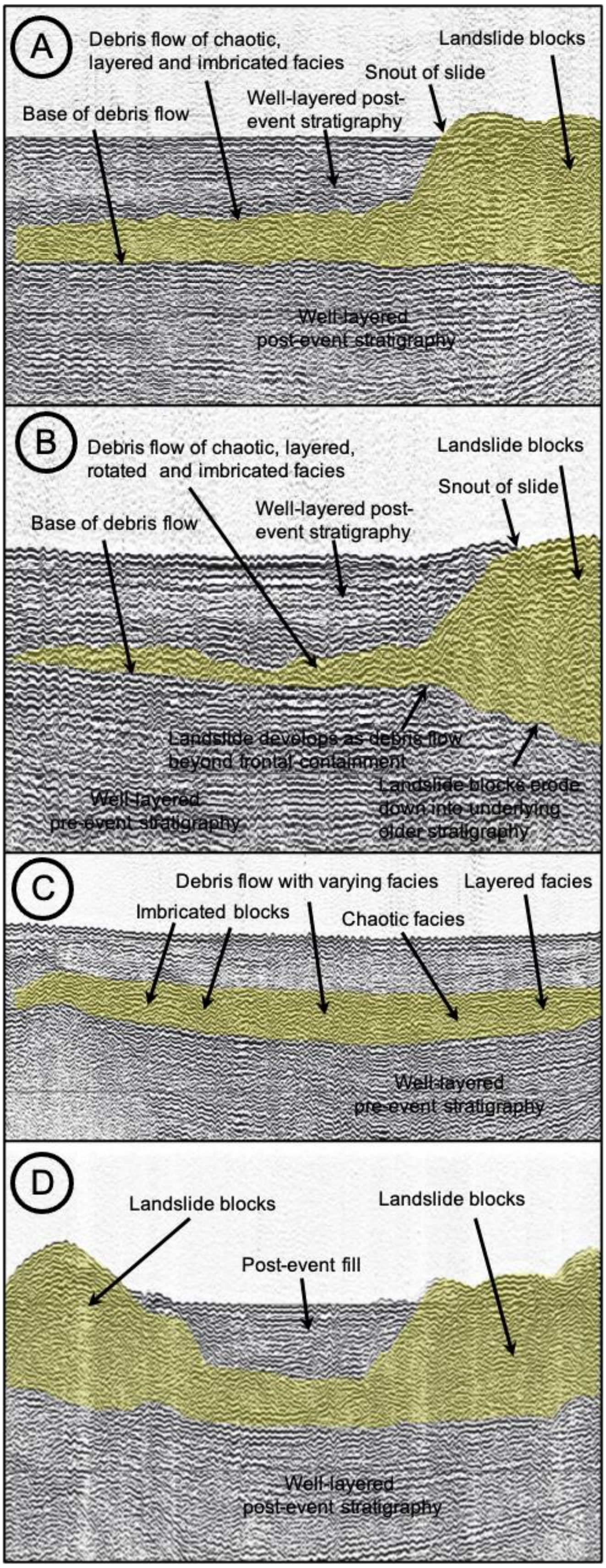

Supplementary Figure 12. Failure plane geometries and volumetric calculations. A) Model of shallow failure scenario with subaerial failure plane projected to a depth of 100-120 m to align to slope features and depth of adjacent basins. B) Model of deep failure scenario with subaerial failure plane projected to caldera basin floor depth. C) Cross sections of proposed end-member failure geometries of the shallow and deep failure planes with estimated area and volumes of failures.

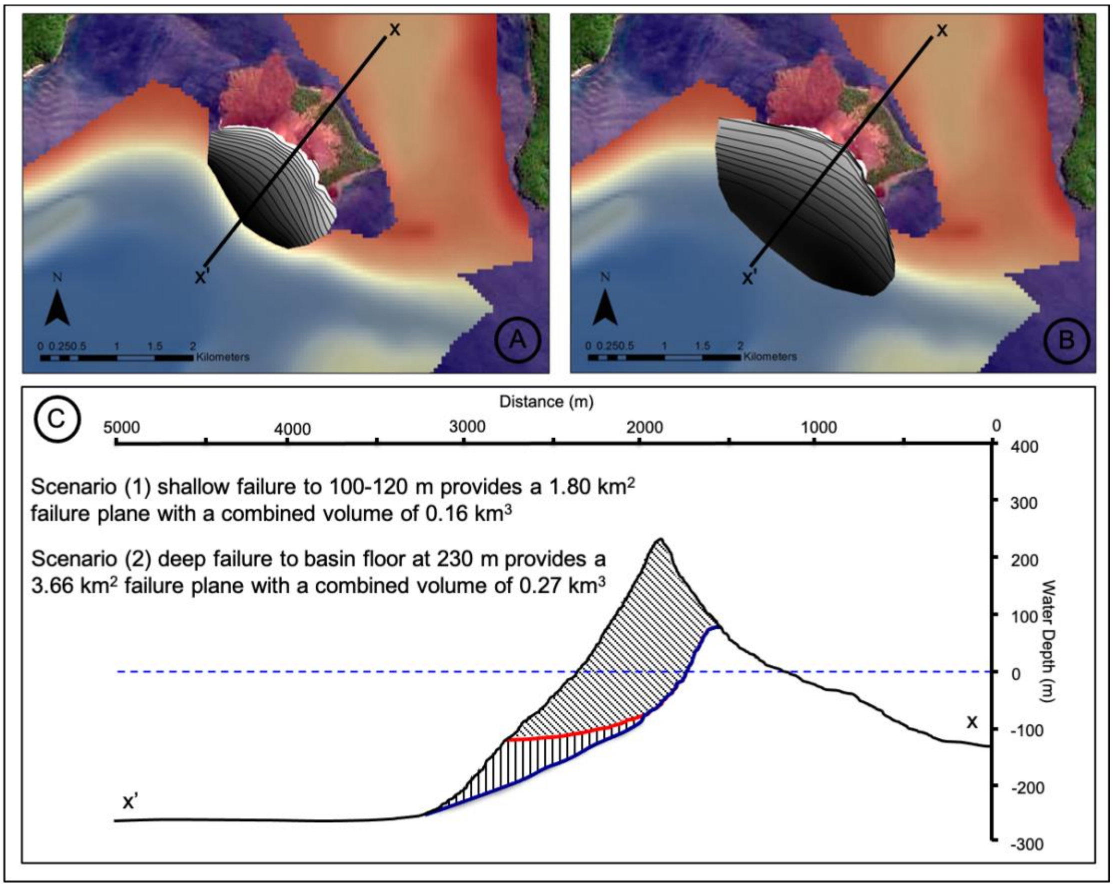

Supplementary Figure 13. Slope profiles and volumetric estimations. A) Slope profile (location on fig. 4A) matching the profile from Muhari et al. (2019) showing the 1990 and 2019 bathymetry and seafloor first-return of seismic reflection line SS-11D (this paper) compared to published bathymetry from 2016 and 2018 surveys (Muhari et al., 2019). B) Slope profiles of the 1990 and 2019 bathymetry with seafloor first-return of seismic reflection line SS-5B (location on fig. 4B); C) Slope profiles of the 1990 and 2019 bathymetry with seafloor first-return of seismic reflection line SS-11C (location on fig. 4B).

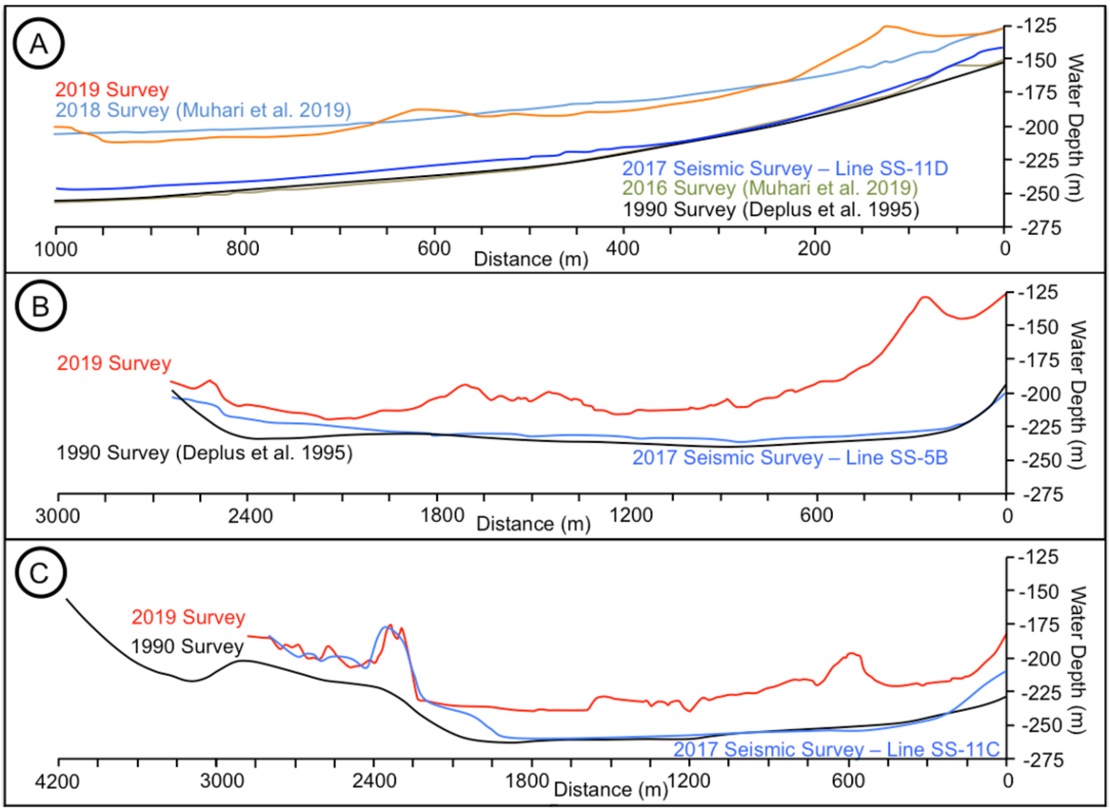

Supplementary Figure 14. Landslide volume estimates of the Anak Krakatau flank collapse from previous works.

Reference*	Subaerial Volume (km^3)	Submarine Volume (km^3)	Total Volume (km^3)	Methods
Giachetti et al. (2012)	-	-	0.28	Flank geometry.
Grilli et al. (2019)	-	-	0.22-0.30	Flank and failure plane geometry.
Williams et al. (2019)	Neg.	0.1	0.1	Flank geometry based upon interpretations of the subaerial limit of the failure. As little as 0.07 km^3 towards tsunamigenesis.
Walter et al. (2019)	0.102	-	-	Subtraction of subaerial digital elevation models.
Gouhier and Paris (2019)	0.0938	-	-	Application of a fault plane interpreted from SAR images applied to a reconstructed 2019 digital elevation model.
Paris et al. (2019)	-	-	0.15	Slope geometries extrapolated from subaerial limitation of the failure from Gouhier & Paris (2019).
Paris et al. (2020)	-	-	0.15	Slope geometries extrapolated from subaerial limitation of the failure from Gouhier & Paris (2019).
Heidarzadeh et al. (2020)	-	-	0.175-0.236	Physical experiments and numerical modelling.
Ye et al. (2020)	-	-	0.2	Seismic data inversion.
Mulia et al. (2020)	-	-	0.24	Slope geometries.
Ren et al. (2020)	-	-	0.2-0.3	Numerical landslide-tsunami modelling.
Zengaffinen et al. 2020	-	-	0.21, 0.28	Numerical landslide-tsunami modelling.
Borrero et al. 2020	-	-	0.2	Numerical landslide-tsunami modelling.
Pakoksung et al. 2020	-	-	0.182	Numerical landslide-tsunami modelling.
Kongko et al. 2020	-	-	0.175	Numerical landslide-tsunami modelling.
Omira et al. 2020	-	-	0.135	Numerical landslide-tsunami modelling.

*references in main text
